# Type 1 Gastric Neuroendocrine Tumor Found on Endoscopic Polypectomy

**DOI:** 10.7759/cureus.4193

**Published:** 2019-03-06

**Authors:** Hector H Gonzalez, Mary Acosta, George Yazji, Matthew Q Bromer

**Affiliations:** 1 Internal Medicine, Florida Atlantic University Charles E. Schmidt College of Medicine, Boca Raton, USA; 2 Family Medicine, North Florida Regional Medical Center/ University of Central Florida College of Medicine, Gainesville, USA; 3 Gastroenterology, Bethesda Hospital East, Boynton Beach, USA

**Keywords:** gastric neuroendocrine tumor, gastric polyp, gastrin, pernicious anemia

## Abstract

Gastric neuroendocrine tumors (GNET) are rare gastric neoplasms accounting for <1% of all gastric neoplasms. The World Health Organization (WHO) categorized these neoplasms as types 1-3 to help predict malignant potential and long-term survival and guide management. Improved outcomes have been shown with endoscopic resections, but further studies are needed to confirm the best approach. We present a case of a 56-year-old woman who demonstrated the classic features of type one GNET with mucosal and submucosal involvement in the setting of primary atrophic gastritis, secondary hypergastrinemia, and underlying pernicious anemia. In general, standardizing treatment has been difficult due to a variable presentation.

## Introduction

Gastric neuroendocrine tumors (GNET) are exceedingly rare, accounting for <1% of gastric neoplasms and 8.7% of gastrointestinal neuroendocrine tumors [1]. In 2010, the World Health Organization (WHO) categorized these neoplasms as grade 1-3, based on the histological classification, Ki-67 index, and mitotic activity [2]. Of the three types of GNET, type one is the most common, making up 70% to 80% of GNET [1-2]. This specific subtype arises in the setting of chronic atrophic gastritis, leading to the inability of the parietal cells to secrete gastric acid. This condition allows for hyperplasia of gastric antral G cells leading to hypergastrinemia. Gastrin binds to the cholecystokinin-2 (CCK-2) receptor on the enterochromaffin-like (ECL) cells to encourage hyperplasia allowing for the development of type one GNET [3]. These neuroendocrine tumors are classically associated with ECL cell hyperplasia, hypergastrinemia, and chronic atrophic gastritis with or without pernicious anemia [4]. It has also been hypothesized that proton pump inhibitors (PPI) may promote GNET formation secondary to PPIs, leading to hypergastrin state. Given the widespread use of PPIs and the rarity of the tumor, there is likely a co-factor involved in the formation that is not yet fully understood [5]. Type two GNETs are also associated with elevated gastrin levels but typically occur with hypertrophic mucosa. Type three GNETs are most likely to be malignant with a higher risk for a metastatic spread at diagnosis. We report a case of GNET arising in a sessile polyp during endoscopic polypectomy.

## Case presentation

**Figure 1:**
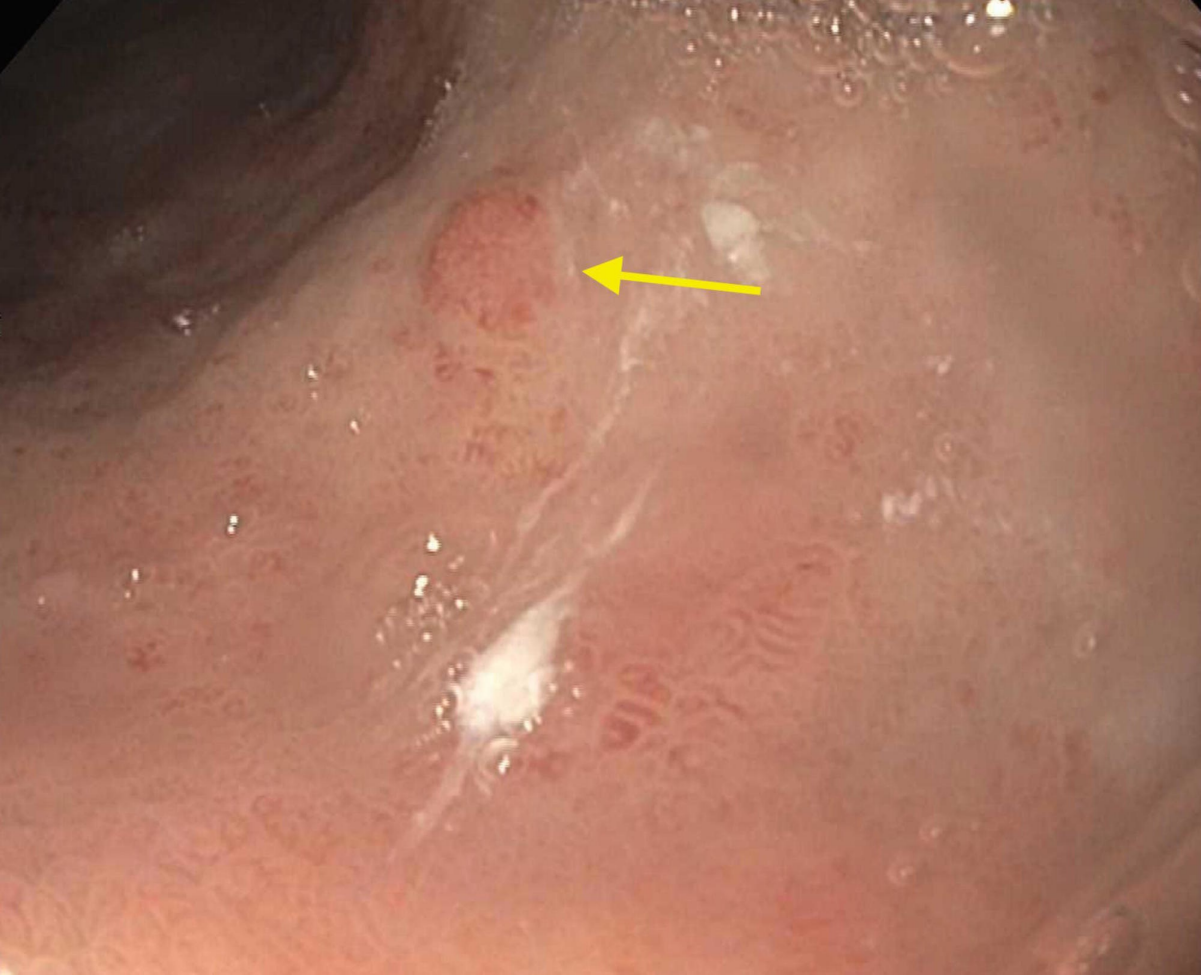
Endoscopic image of sessile polyp found in the gastric body (yellow arrow)

**Figure 2:**
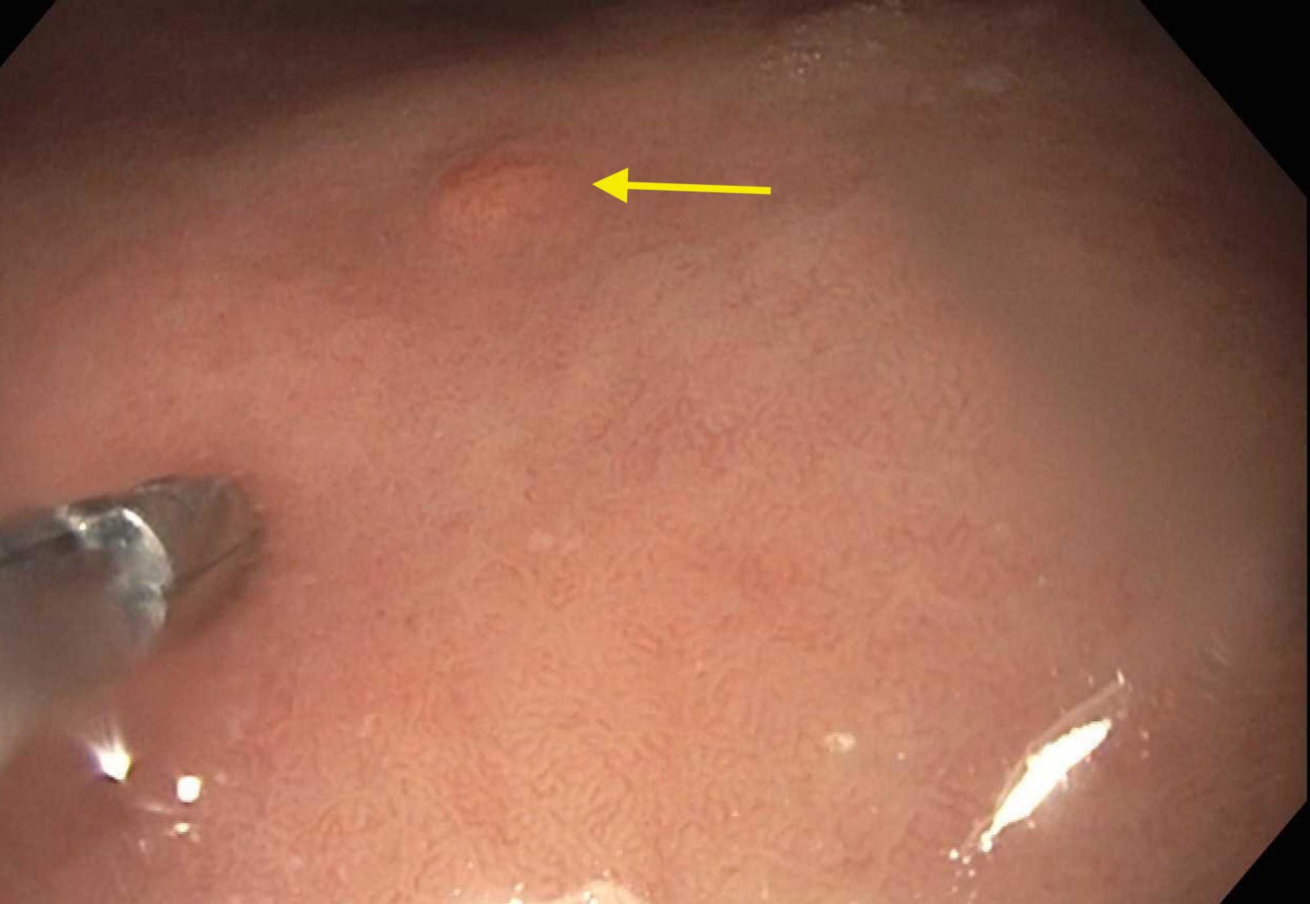
Endoscopic view of gastric polyp (yellow arrow) with biopsy forceps for size comparison

A 56-year-old woman with a history of cholelithiasis and irritable bowel syndrome presented to the office for postprandial, colicky left upper quadrant pain radiating to the right shoulder lasting approximately 45 minutes. The pain was associated with three to four episodes of diarrhea and dyspepsia. Lab studies showed gastrin level off PPI of 2100 pg/mL. Ultrasound of abdomen showed diffusely increased hepatic echogenicity suggesting fatty change and cholelithiasis. The patient was sent for a hepatobiliary iminodiacetic acid (HIDA) scan that was performed, showing an ejection fraction of 90%. Elective cholecystectomy was scheduled and performed without resolution of the symptoms. The patient was sent for colonoscopy that was negative. Esophagogastroduodenoscopy (EGD) with biopsy and snare polypectomy showed erythematous “carpet-like” atrophic mucosa in the antrum, five to six sessile polyps (the largest being 10mm) with nodular mucosa in the body of stomach and fundus (Figures 1-2). 

**Figure 3:**
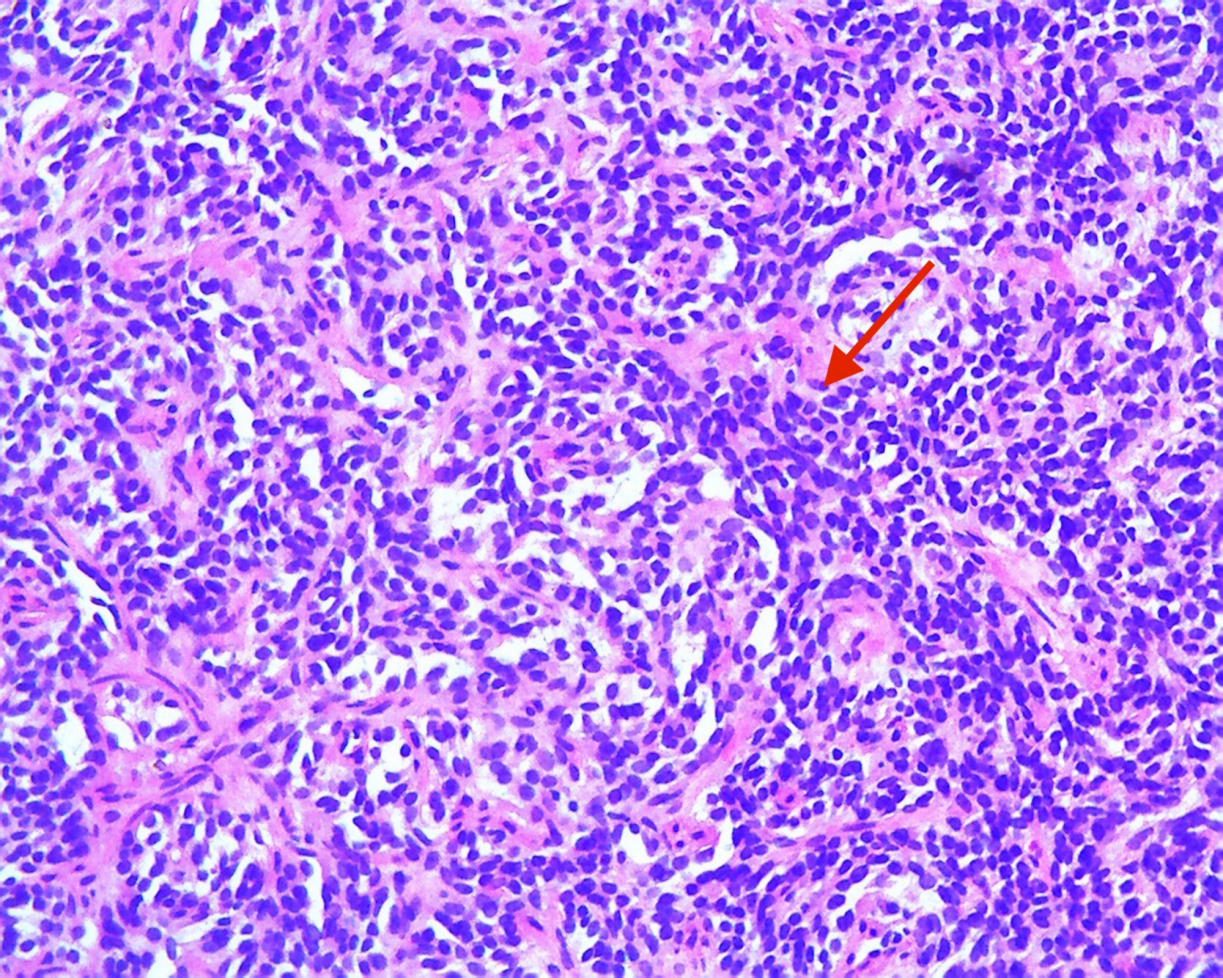
Histopathology slide showing gastric neuroendocrine tumor cells (red arrows)

**Figure 4:**
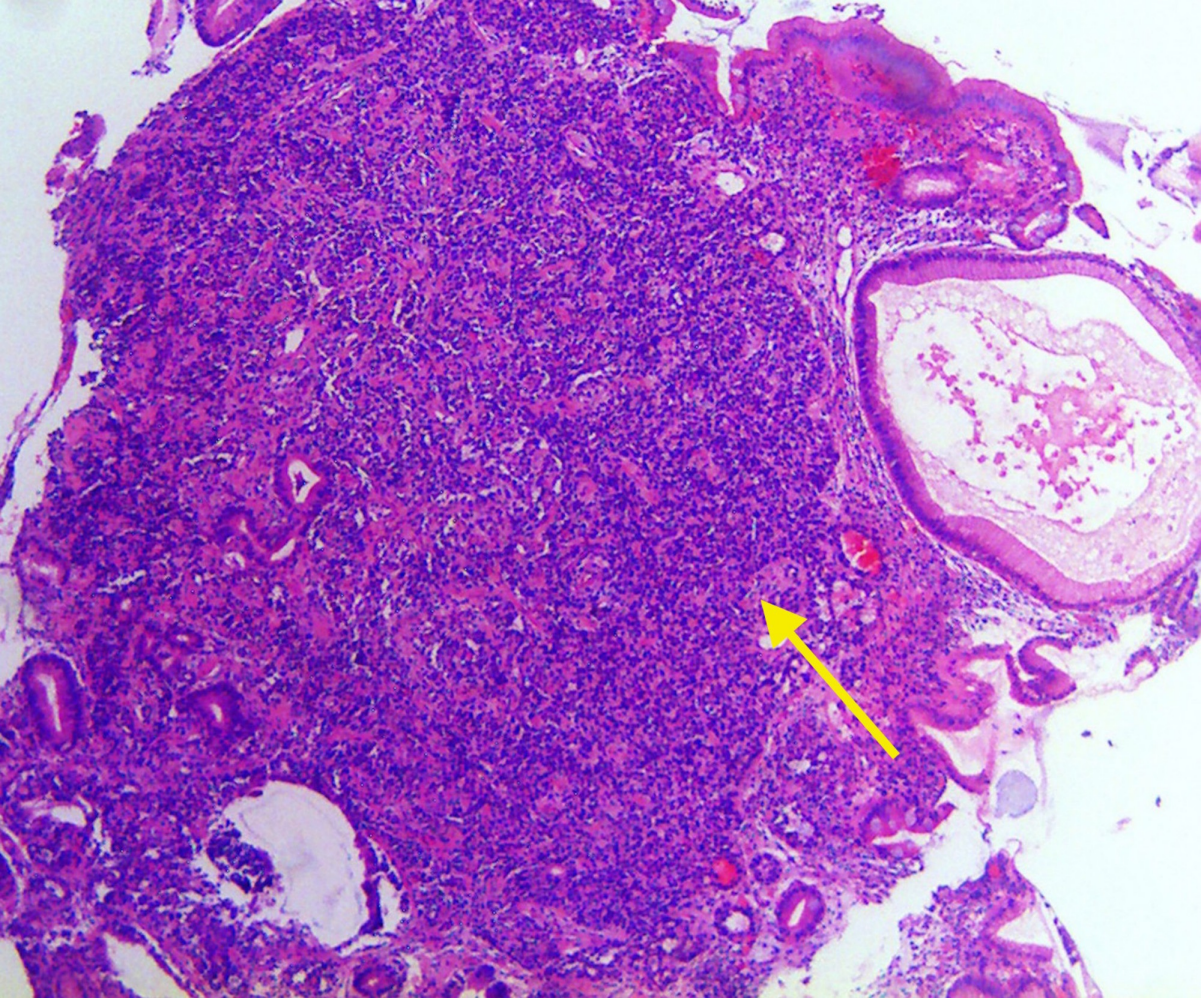
Hematoxylin and eosin stain (100x) of gastric body showing gastric neuroendocrine tumor cells (yellow arrow)

Endoscopic ultrasound demonstrated a 2.5 x 1.5-mm isoechoic mass in the body of the stomach with invasion into the submucosa. Octreotide scan was negative for other organ involvement. Histology from gastric polyps revealed grade 2 well-differentiated GNET involving the mucosa and submucosa (Figures 3-4). 

**Figure 5:**
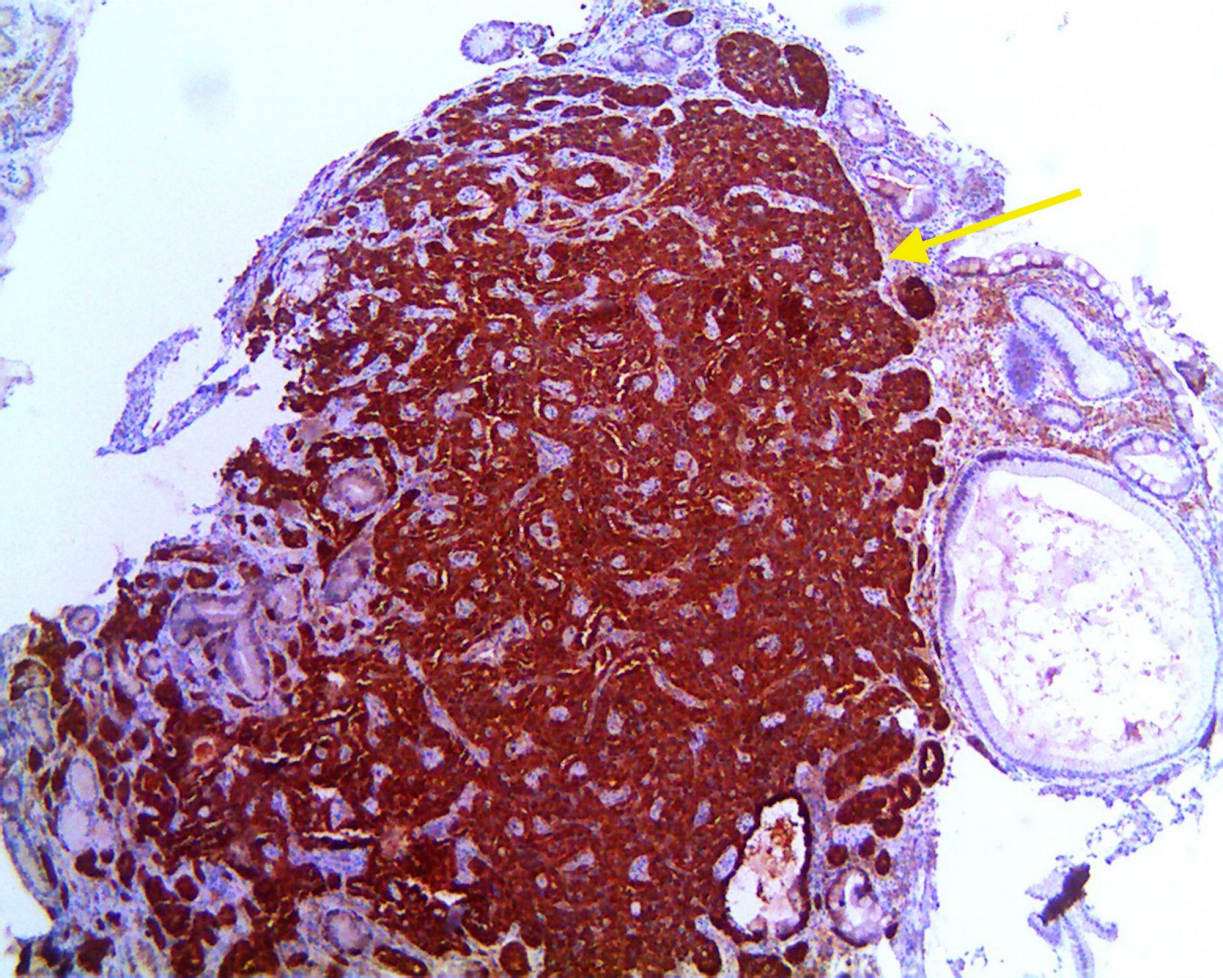
Immunohistochemistry demonstrating positive chromogranin stain (yellow arrow)

**Figure 6:**
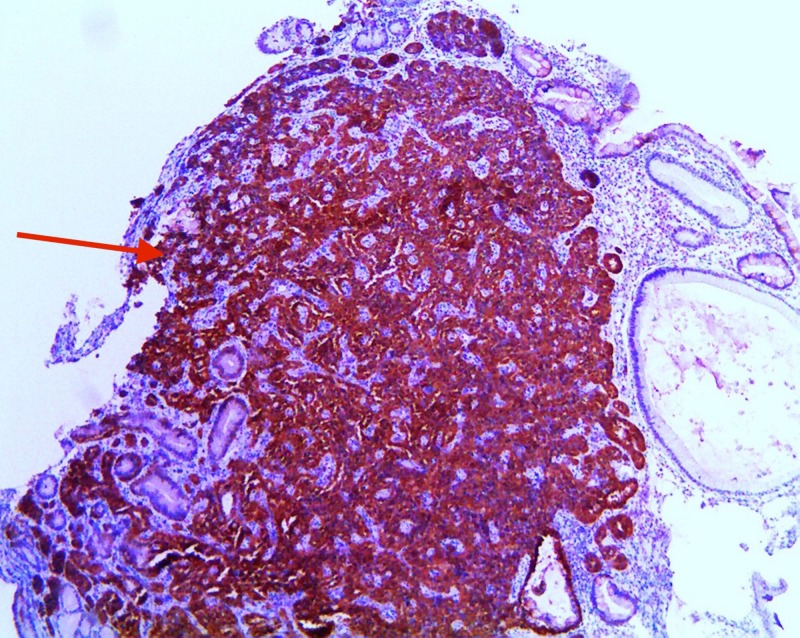
Immunohistochemistry demonstrating positive synaptophysin stain (red arrow)

Pathology also showed +Ki-67 in 3.8% of tumor cells with mitotic activity 0.4/10 high-power fields. Immunohistochemistry of the sample showed +chromogranin, + synaptophysin, +CD56, pankeratin, and focal CDX2+ (Figures 5-6).

The patient was treated with endoscopic resection, pantoprazole 40 mg daily, vitamin B12, iron supplementation, and follow-up EGD in six months.

Follow-up endoscopy at six months with ultrasound has shown multiple mucosal polyps up to 10 mm in the body and fundus. Biopsies of these lesions show well-differentiated carcinoid tumors and chronic gastritis with intestinal metaplasia.

## Discussion

GNETs have historically been divided into two pathological subgroups based on the serum gastrin level. More recently, this subdivision has been further classified into three distinct subgroups: type one, type two, and type three. Types one and two are similar in that they develop from ECL cells and present with elevated gastrin levels [1,3-4]. Type one GNET often is associated with chronic atrophic gastritis with low stomach acid levels as opposed to type two GNET that is associated with hypertrophic gastropathy and elevated stomach acid levels [1-5]. Type three GNET is made up of poorly differentiated cells with high rates of malignancy as well as metastasis at the time of diagnosis [6]. Type two can be associated with multiple endocrine neoplasia type one (MEN 1) or Zollinger Ellison syndrome [4-6]. Of the three subsets, type one GNETs are the most common comprising 55% to 80% of ECL neuroendocrine tumors [1,3-5]. It typically presents as multiple polyps on atrophic gastric mucosa. They tend to be small in size measuring 0.5 to 1 cm with only a 5% lymph node metastasis rate [2]. The risk of lymph node metastasis increases when the lesion is greater than 2 cm and has infiltrated the muscularis propia [7]. Type two GNETs are less common, but also present as multiple polyps on hypertrophic gastric mucosa. They are typically <2 cm when diagnosed and are associated with elevated gastrin levels and have a lymph node metastasis rate of 30% [5-8]. Type three is also less common and is more likely to present as a single, sporadic tumor with normal mucosa. They are typically >2 cm when discovered and have a lymph node metastasis percentage of 70%, often being discovered once the tumor has metastasized [2].

Gastric polyps are often found incidentally on endoscopy. A one-year study conducted in the United States on 120,000 patients showed a 6% prevalence of gastric polyps found on upper endoscopy [8]. When polyps are found on endoscopy, biopsy or excision will aid in a histologic diagnosis. Most polyps, about 70% to 90%, are hyperplastic polyps or fundic polyps [5-8]. Hyperplastic polyps are asymptomatic, found incidentally, rarely carry malignant risk, and occur in the setting of chronic inflammatory conditions or *H. pylori* infection [5-8]. In the United States and other western countries, fundic gland polyps are most common due to common PPI use and low prevalence of *H. pylor*i infection. These also are unlikely to progress to malignancy. Adenomas, although rare, have malignant potential and should be excised completely with endoscopic surveillance one year later [6-9]. GNET types one and two are typically seen as multiple, broad-based, firm yellowish lesions in the fundus and body of the stomach. Type three is usually seen as single tumors in the antrum. Biopsy results often show prominent solid nest or insular pattern as they arise from the ECL cells [1,3-6]. Current guidelines suggest polyp biopsy and resection when there are small solitary polyps [1-9]. If the polyp is >1 cm in diameter or is known to be neoplastic, polypectomy should be performed [6-9]. Mucosa should also be sampled to rule out atrophic gastritis and *H. pylori* infection when polyps are discovered on endoscopy [9].

Pathologic evaluation of biopsied GNET is graded based off of mitotic count and Ki-67 labeling. Grade 1 is a mitotic count of less than two per 10 high power fields (HPF) and/or less than 3% Ki-67 labeling index, which is expressed during the active phases of the cell cycle. Grade 2 has a mitotic count of two to 20 per 10 HPFs and/or 3% to 20% Ki-67 labeling index. Grade 3 tumors are high-grade neuroendocrine carcinomas [1,3-8]. If there is a discrepancy between the mitotic count and the Ki-67 index, a higher grade is used [2]. Chromogranin A (CgA), similar to the other granins, is an acidic protein that makes up secretory granules of the neuroendocrine cells [1,5-6]. Types one and two GNETs are CgA positive, while type three GNETs are negative. In addition to tissue staining, CgA can also be measured in the serum or plasma. False positives in the serology can occur in chronic atrophic gastritis and with PPI use. It can also be affected by multiple chronic medical conditions and changes in diet or exercise [1]. Thus, CgA is not recommended as a screening tool but rather should be utilized when there is a high pretest probability. Neuroendocrine tumors also stain positively for synaptophysin, which is involved in secretory vesicles of neuronal cells, and CD56 [1-8]. Chromogranin is thought to be more specific, while synaptophysin is the more sensitive marker. It has also been noted that CgA expression may be weak in poorly differentiated tumors, but synaptophysin expression is usually intact even in poorly differentiated tissues. Keratin staining and CDX 2 staining are done to ensure the absence of paragangliomas as was done in the above case [10].

Both anatomic and functional imaging can be helpful when diagnosing neuroendocrine tumors of unknown primary location or unknown metastatic disease. Computed tomography (CT) scan is a good imaging modality for the lungs and CT enterography can detect neuroendocrine tumors (NET) as small as 0.5 cm [11]. Magnetic resonance imaging (MRI), specifically T2 weighted images, can be helpful in identifying metastatic disease in the liver [11]. Functional imaging studies are helpful in neuroendocrine tumor evaluation. Gadolinium (Ga) contrast can show a temporal relationship between tumor and normal liver uptake. Octreotide scan study allows for whole body evaluation of tissue with somatostatin receptors that can help identify metastatic tumors for tissues expressing somatostatin receptor subtypes 2 and 5 [1-11]. Sensitivity and specificity of octreotide scan can be close to 100% for tumors >1 cm, but cannot detect NETs smaller than that size [11]. It has also suggested that this scan can predict clinical response to somatostatin analogs. Ga-68 somatostatin analog (SA) positron emission tomography (PET) scanning was subsequently developed and can detect tumors as small as 3-6 mm [11]. It has also been shown that Ga-68 SA PET has higher sensitivity for NET as compared to octreotide scans. Given its effectiveness, practitioners are often limited by cost and geographic availability so octreotide scans are still the first-line NET imaging choice. Those patients where Ga-68 SA PET would be more useful include those with metastatic disease of unknown origin, those being evaluated for liver transplant if there is a question of extrahepatic lesions, patients with small, suspicious lesions, and those with biochemical evidence, but no anatomic findings of NET [11]. Fluorodeoxyglucose (FDG)-positron emission tomography (PET) is rarely helpful for NET as it measures glucose uptake, and most NETs do not have high glucose affinity [11]. Types one and two gastric NETs without lymph node involvement by anatomic imaging likely do not require functional imaging [11].

Our case demonstrated type one GNET with submucosal involvement in a patient with primary atrophic gastritis and underlying pernicious anemia. About 65% of patients with type one GNET will present with pernicious anemia such as the case with this presentation [12]. It has been shown that in patients with pernicious anemia there is an increased risk (about two- to three-fold increase) for gastric cancer in the setting of chronic atrophic gastritis [13-14]. The risk of GNET in the setting of pernicious is significant enough for current recommendations to perform a single endoscopy to identify potential for lesions [1-14]. There is not enough data at this time to support routine endoscopic surveillance in patients with pernicious anemia [9]. Aside from the lab values showing pernicious anemia with low B12 levels as well as elevated gastrin levels, this tumor is often asymptomatic or presents with nonspecific abdominal pain. This patient initially presented with colicky right upper quadrant abdominal pain, which is not a characteristic finding and prompted elective cholecystectomy without resolution of symptoms. Given the persistence of symptoms as well as pernicious anemia in this patient, endoscopy was performed and demonstrated multiple polyps. Pathologic evaluation found well-differentiated neuroendocrine tumor cells with grade 2 distinction by Ki-67 index. Pathology confirmed neuroendocrine origin with positive chromogranin A, synaptophysin, and CD56. As commonly seen in type one GNET, the patient had multiple polyps (Poster: Gonzalez H MD, Yazji G MD, Acosta M BS. Bromer M DO. A Case of Type I Gastric Neuroendocrine Tumor Found on Endoscopic Polypectomy. ACG annual meeting. 2018.).

Treatment of gastric neuroendocrine tumors is stratified by type, as the prognosis varies greatly from types one to three tumors. The standardization of treatment of type one GNET has been difficult due to the lack of consensus on diagnostic histological classification and a typically benign disease course. Treatment modalities include endoscopic lesion resection, antrectomy, and pharmacological therapy [9-14]. Typically, lesions less than 1 cm are removed by endoscopic resection [9-14]. The literature clearly suggests improved outcomes with endoscopic resections [9-14]. For example, Ugyun et al. showed that endoscopic follow-up in 22 patients was safe and effective with a recurrence rate of 18% [3]. Endoscopic follow-up typically occurs between six and 12 months [2]. A second study of 16 patients with type one GNET showed that 100% of the type one GNETs biopsied were localized to the stomach mucosa and were benign [1-3]. They were treated with local excision, partial resection, and total gastrectomy. One of the nine patients treated with local excision had a recurrence of type one GNET, while eight had continued atrophic gastritis on repeat endoscopy [6]. Cases have shown that antrectomy significantly and persistently decreases the gastrin levels which in theory removes the stimulus for GNET formation [15]. Further comparative studies of treatment modalities including follow-up with endoscopy versus surgery need to be conducted. Somatostatin analogs, specifically octreotide, have also been explored as they lower serum gastrin levels blocking the proposed pathogenesis of ECL hyperplasia [8-16]. One case report utilized both endoscopic resection and sandostatin treatment for a patient with multiple recurrent lesions and demonstrated no recurrence at 24 months after intermittent use of sandostatin for the two years prior with endoscopic follow-up every six months [16]. 

## Conclusions

This case highlights the rise in type one GNETs found incidentally on endoscopy. Our case is atypical in the presentation as the patient initially complained of right upper quadrant postprandial, colicky pain. Post-cholecystectomy, the patient had persistent symptoms prompting endoscopy in the setting of pernicious anemia. Treatment modalities are not well delineated, but endoscopic resection in small polyps have shown to be associated with low risk of recurrence. Given this, our patient had the recurrence of polyps at six months. More comprehensive studies on treatment modalities will be needed to ensure optimal curative intervention.
